# Selank Administration Affects the Expression of Some Genes Involved in GABAergic Neurotransmission

**DOI:** 10.3389/fphar.2016.00031

**Published:** 2016-02-18

**Authors:** Anastasiya Volkova, Maria Shadrina, Timur Kolomin, Lyudmila Andreeva, Svetlana Limborska, Nikolay Myasoedov, Petr Slominsky

**Affiliations:** ^1^The Department of Molecular Basis of Human Genetics, Institute of Molecular Genetics Russian Academy of SciencesMoscow, Russia; ^2^The Department of Chemistry of Physiologically Active Compounds, Institute of Molecular Genetics Russian Academy of SciencesMoscow, Russia

**Keywords:** Selank, GABA, glyproline, regulatory peptide, gene expression

## Abstract

Clinical studies have shown the similarity of the spectrum of physiological effects of Selank and classical benzodiazepines, such as diazepam and phenazepam. These data suggest that there is a similar basis of their mechanism of action. To test this hypothesis we studied the effect of Selank and GABA on the expression of genes involved in neurotransmission. We analyzed the expression of 84 genes involved in neurotransmission (e.g., major subunit of the GABA receptor, transporters, ion channels, dopamine, and serotonin receptors) in the frontal cortex of rats 1 and 3 h after the administration of Selank or GABA (300 μg/kg) using real-time PCR method. We found significant changes in the expression of 45 genes 1 h after the administration of the compounds. Three hours after Selank or GABA administration, 22 genes changed their expression. We found positive correlation between the changes in genes expression within 1 h after administration of Selank or GABA. Our results showed that Selank caused a number of alterations in the expression of genes involved in neurotransmission. The data obtained indicate that Selank is characterized by its complex effects on nerve cells, and one of its possible molecular mechanisms is associated with allosteric modulation of the GABAergic system.

## Introduction

Regulatory peptides play key roles in the formation, development, and normal functioning of the nervous system. They are not understood fully despite the accumulating experimental data in recent years. The study of their mechanisms of action is of particular interest because regulatory peptides have potential in the creation of safe drugs on their basis with specific clinical properties and direct physiological effects. One representative of this class of drugs is the synthetic regulatory peptide Selank. It was designed and produced at the Institute of Molecular Genetics, Russian Academy of Sciences, in cooperation with the V.V. Zakusov Research Institute of Pharmacology, Russian Academy of Medical Sciences. Selank is a synthetic analog of the endogenous tuftsin molecule (the short Thr-Lys-Pro-Arg fragment of the human immunoglobulin G heavy chain), which was elongated at the C terminus via the addition of three natural L-amino acids (Pro-Gly-Pro) to improve its metabolic stability and yield a relatively longer duration (Ashmarin et al., 2005; Ashmarin, 2007).

Selank has pronounced anxiolytic activity and acts as a stable neuropsychotropic, antidepressant, and antistress drug that relieves aggression and fear reaction in different animal species (Kozlovskii and Danchev, 2002; Sollertinskaya et al., 2008; Semenova et al., 2010). Selank also has a nootropic action, which positively influences the formation of memory and learning processes (Kost et al., 2001; Sokolov et al., 2002; Semenova et al., 2007, 2009; Narkevich et al., 2008), and marked immunomodulatory activity (Uchakina et al., 2008; Ershov et al., 2009; Andreeva et al., 2010).

Clinical studies have shown that the effect of Selank is similar to that of tranquilizers at low doses, but is not accompanied by the unwanted side effects of benzodiazepine tranquilizers such as amnesia, withdrawal, and dependence (Seredenin et al., 1990, 1998). Benzodiazepines are allosteric modulators of the type-A γ-aminobutyric acid receptor (GABA_A_R) and can increase the inhibitory action of GABA, the major inhibitory neurotransmitter in the CNS. The similarity of the spectrum of physiological effects of Selank and classical benzodiazepines (such as diazepam and phenazepam) suggests that there is a similar basis of their mechanism of action; that is, the allosteric modulation of GABA_A_ receptors.

Previously, it was shown that in the presence of Selank the amount of the specifically bound ligand, [^3^H] GABA, varied, and preliminary intranasal administration of peptide also induced changes in the number of specific binding sites of [^3^H] GABA but did not affect the affinity of the receptors (V'Yunova et al., 2014). Based on these data, the authors suggested that Selank can lead to a rapid change in the state of the GABAergic system by binding the peptide to GABA receptors and, thus, allosterically modulating the activity of GABA_A_ receptor.

In this study, we evaluated the contribution of the GABAergic system to the molecular mechanism responsible for the anxiolytic action of Selank. To test the hypothesis that Selank acts through GABA_A_ receptors, we investigated its effect on changes in the mRNA levels of the genes encoding the major subunits of the GABA receptors, transporters and ion channels involved in the transport of GABA, and those of other proteins involved in neurotransmission in rat brain 1 and 3 h after administration of the peptide. To identify the effects associated with the activation of GABA_A_ receptors, we analyzed the changes in the expression of the investigated genes in response to the action of the primary ligand, GABA.

## Materials and methods

### Chemicals

Dry preparations of Selank (Nα-Thr-Lys-Pro-Arg-Pro-Gly-Pro-Diacetate Salt) and GABA (γ-aminobutyric acid) were dissolved to a concentration of 10 mg/ml in deionized water.

### Animal model

The male Wistar rats with an average weight of 200 g were used in the experiment. The animals were kept under the standard conditions with free access to water and food, and a 12 h light/dark cycle. The animals (*n* = 30) were divided into three groups: one control group (*n*_1_ = 10) and two experimental groups: Selank group (*n*_2_ = 10) and GABA group (*n*_3_ = 10). A single intranasal administration of the water solution of Selank or GABA was performed on each animal from the experimental groups (6 μl at the concentration of 300 μg to 1 kg of body weight) and the equivalent volume of deionized water was performed on each animal from the control group. Selank dose of 300 μg/kg was selected based on the data that this dose was the most effective therapy dose exerting anxiolytic action (Seredenin et al., 1998; Kozlovskaya et al., 2003). The first half of animals from each group was decapitated 1 h after the administration of the compounds, the second half—after 3 h. Immediately after the decapitations, the rat frontal cortexes were dissected, placed into sterile test tubes (free of RNase and DNase), and frozen in liquid nitrogen with subsequent storage at −70°C.

The animal experiments were carried out in accordance with the National Institutes of Health Guide for the Care and Use of Laboratory Animals (NIH publication N_o_ 80-23) and the statement of the ethics committee of the Institute of Molecular Genetics, Russian Academy of Sciences.

### RNA isolation and reverse transcription

Frontal cortex tissues obtained from each rat were pooled according to the corresponding groups and time points, resulting in six pools. Total RNA was extracted from each resulting pool of tissues using the RNeasy® Mini Kit (Qiagen, Germany) according to the manufacturer's protocol. First-strand cDNAs were synthesized using the RT^2^ First Strand Kit (Qiagen, Germany) according to the manufacturer's protocol.

### Real-time quantitative RT-PCR

The effect of Selank and GABA on the expression of genes was studied with the help of the real-time PCR method using a Custom RT^2^ Profiler™ PCR Array: CAPR11632 (Qiagen, Germany). Amplification was carried out on the device StepOnePlus™ Real-Time qPCR System (Life Technologies, USA) using the RT^2^ SYBR Green Mastermixes (Qiagen, Germany). Thermal cycling was carried out as follows: (1) 95°C for 600 s, followed by (2) 40 cycles of 15 s at 95°C and 60 s at 60°C. All reactions were repeated three times in each group for each time point.

### Statistical analyses

The threshold reaction cycle (Ct) values obtained for the genes under study were normalized to the *Ct*-values of the reference genes. Statistical data analysis of the normalized *Ct*-values was performed using the Relative Expression Software Tool 2009 (REST 2009) v.2.0.13, and Statistica 8.0. Genes with significant changes (*p* ≤ 0.05) in the mRNA level by 1.5 times or more were considered in the analysis to assess the changes in expression by the action of the test compounds.

## Results

We studied the effects of Selank and GABA on changes in the mRNA levels of 84 genes involved in neurotransmission in the frontal cortex of rats 1 and 3 h after the intranasal administration of the compounds. The intranasal administration of Selank was shown to be optimal for delivery of peptide molecules in the CNS (Zolotarev et al., 2006; Ashmarin et al., 2008).

Preliminary analysis showed that, among the 84 studied genes, seven genes (*Csf2, Drd4, Htr3b, Il2, Mmp7, Mmp10*, and *Npffr2*) had a high threshold reaction cycle (*Ct* > 35), indicating a low content of mRNA in the tissue. Therefore, these genes were excluded from further analysis.

Of the remaining 77 genes, summarily 45 genes showed changes in mRNA level 1 h after Selank or GABA administration (Table 1). Twenty-five, or more than half of these 45 genes, showed changes in mRNA level after administration of either compound: *Abat, Adcy7, Adora1, Bcl2l1, Cacna1a, Cacna1b, Cx3cl1, Drd3, Drd5, Gabrb3, Gabre, Gabrq, HcRt, Hcrtr2, Htr3a, Myc, Npffr1, Nsf, P2rx7, Prlhr, Slc32a1, Slc38a1, Slc6a1, Slc6a11*, and *Slc8a3*. The mRNA level of four genes (*Drd1a, Drd2, Ptgs2*, and *Slc6a13*) changed only after Selank administration, and that of 16 genes (*Aldh5a1, Birc3, Birc5, Ccnd1, Egr1, Gabbr1, Gabra1, Gabrb1, Gabrd, Gabrg3, Gad1, Glul, Htr1b, Jun, Junb*, and *Slc6a12*) changed only after GABA administration.

**Table 1:** **The relative mRNA levels of genes involved in neurotransmission in rat frontal cortex one and three hours after the administration of Selank or GABA (the table lists only the genes which showed a statistically significant change in mRNA levels)**.

	**Gene symbol**	**Official full name**	**1 h**				**3 h**			
			**Selank**		**GABA**		**Selank**		**GABA**	
			**Fold change**	***p*-value**	**Fold change**	***p*-value**	**Fold change**	***p*-value**	**Fold change**	***p*-value**
Subunits of the GABA receptors	*Gabbr1*	Gamma-aminobutyric acid (GABA) B receptor 1	0.74	0.0111	0.57^*^	0.0002	1.36	0.0013	1.19	0.0247
	*Gabra1*	Gamma-aminobutyric acid (GABA) A receptor, alpha 1	1.33	0.0531	1.94^*^	0.0002	0.73	0.0015	0.93	0.1772
	*Gabra6*	Gamma-aminobutyric acid (GABA) A receptor, alpha 6	1.25	0.4384	1.55	0.0968	0.83	0.1942	7.56^*^	0.0004
	*Gabrb1*	Gamma-aminobutyric acid (GABA) A receptor, beta 1	1.09	0.1980	1.57^*^	0.0005	0.94	0.0970	0.82	0.0096
	*Gabrb3*	Gamma-aminobutyric acid (GABA) A receptor, beta 3	1.58^*^	0.0006	2.07^*^	0.0009	0.86	0.0211	0.95	0.3392
	*Gabrd*	Gamma-aminobutyric acid (GABA) A receptor, delta	0.81	0.0366	0.37^*^	0.0015	0.88	0.0807	1.20	0.0479
	*Gabre*	Gamma-aminobutyric acid (GABA) A receptor, epsilon	0.05^*^	0.000009	0.06^*^	0.000008	16.10^*^	0.0002	1.06	0.7443
	*Gabrg3*	Gamma-aminobutyric acid (GABA) A receptor, gamma 3	1.29	0.0147	1.95^*^	0.0009	0.98	0.5120	1.00	0.9307
	*Gabrq*	Gamma-aminobutyric acid (GABA) receptor, theta	0.05^*^	0.000019	0.05^*^	0.00002	13.30^*^	0.00001	1.21	0.2129
Dopamine receptors	*Drd1a*	Dopamine receptor D1A	1.98^*^	0.0037	0.89	0.0762	1.36	0.0003	1.76^*^	0.0011
	*Drd2*	Dopamine receptor D2	1.60^*^	0.0002	1.30	0.0001	1.11	0.0040	1.46	0.0001
	*Drd3*	Dopamine receptor D3	3.36^*^	0.0047	1.96^*^	0.0061	0.83	0.1832	0.57^*^	0.0144
	*Drd5*	Dopamine receptor D5	0.40^*^	0.0065	0.25^*^	0.0015	1.59^*^	0.0293	0.93	0.6347
Serotonin receptors	*Htr1b*	5-hydroxytryptamine (serotonin) receptor 1B	0.83	0.1542	0.58^*^	0.0004	1.59^*^	0.0090	1.57^*^	0.0145
	*Htr3a*	5-hydroxytryptamine (serotonin) receptor 3a	0.52^*^	0.0019	0.38^*^	0.0011	1.66^*^	0.0036	1.13	0.1647
Ion channels	*Cacna1a*	Calcium channel, voltage-dependent, P/Q type, alpha 1A subunit	0.58^*^	0.0005	0.43^*^	0.000003	1.09	0.2884	1.06	0.4953
	*Cacna1b*	Calcium channel, voltage-dependent, N type, alpha 1B subunit	0.64^*^	0.0004	0.33^*^	0.0086	1.35	0.0068	1.08	0.0782
	*P2rx7*	Purinergic receptor P2X, ligand-gated ion channel, 7	0.53^*^	0.0004	0.22^*^	0.000012	1.40	0.0207	1.27	0.039947
Transporters	*Slc32a1*	Solute carrier family 32 (GABA vesicular transporter), member 1	0.50^*^	0.0025	0.26^*^	0.0001	1.82^*^	0.0011	1.32	0.0299
	*Slc38a1*	Solute carrier family 38, member 1	0.65^*^	0.0010	0.57^*^	0.0001	1.22	0.0177	1.07	0.3890
	*Slc6a1*	Solute carrier family 6 (neurotransmitter transporter, GABA), member 1	0.41^*^	0.0010	0.20^*^	0.0001	1.53^*^	0.0015	1.29	0.0696
	*Slc6a11*	Solute carrier family 6 (neurotransmitter transporter, GABA), member 11	0.29^*^	0.000032	0.18^*^	0.000008	2.29^*^	0.000018	1.05	0.5046
	*Slc6a12*	Solute carrier family 6 (neurotransmitter transporter, betaine/GABA), member 12	1.31	0.0615	0.39^*^	0.0024	0.99	0.9666	0.80	0.2473
	*Slc6a13*	Solute carrier family 6 (neurotransmitter transporter, GABA), member 13	1.97^*^	0.0002	0.69	0.0041	1.04	0.1453	0.88	0.0041
	*Slc8a3*	Solute carrier family 8 (sodium/calcium exchanger), member 3	0.55^*^	0.0036	0.43^*^	0.0007	1.67^*^	0.0006	1.44	0.0055
Other genes involved in neurotransmission	*Abat*	4-aminobutyrate aminotransferase	0.40^*^	0.0001	0.27^*^	0.0001	1.65^*^	0.0019	1.35	0.0493
	*Adcy7*	Adenylate cyclase 7	0.25^*^	0.0013	0.16^*^	0.0009	3.21^*^	0.0004	1.23	0.0073
	*Adora1*	Adenosine A1 receptor	0.57^*^	0.0011	0.40^*^	0.0001	1.36	0.0077	1.47	0.0046
	*Adora2a*	Adenosine A2a receptor	1.44	0.0026	0.73	0.0149	1.51^*^	0.0002	2.06^*^	0.000014
	*Aldh5a1*	Aldehyde dehydrogenase 5 family, member A1	0.68	0.0003	0.56^*^	0.0001	1.22	0.0080	0.99	0.9439
	*Bcl2l1*	Bcl2-like 1	0.63^*^	0.0005	0.50^*^	0.000001	1.32	0.0010	1.11	0.0627
	*Birc3*	Baculoviral IAP repeat-containing 3	1.27	0.0716	0.40^*^	0.0253	1.33	0.1432	0.93	0.8034
	*Birc5*	Baculoviral IAP repeat-containing 5	1.25	0.0653	1.93^*^	0.0008	0.73	0.000047	0.80	0.0019
	*Ccnd1*	Cyclin D1	0.70	0.0092	0.43^*^	0.0006	1.72^*^	0.0001	1.34	0.0235
	*Cx3cl1*	Chemokine (C-X3-C motif) ligand 1	0.56^*^	0.0073	0.24^*^	0.0003	1.42	0.0173	1.50^*^	0.0200
	*Egr1*	Early growth response 1	1.17	0.0230	0.66^*^	0.0103	1.10	0.0145	1.21	0.0053
	*Gad1*	Glutamate decarboxylase 1	0.78	0.0021	0.53^*^	0.0001	1.21	0.0072	1.15	0.0390
	*Glul*	Glutamate-ammonia ligase (glutamine synthetase)	1.48	0.0014	1.72^*^	0.0008	0.82	0.0148	0.95	0.4578
	*HcRt*	Hypocretin	0.04^*^	0.000001	0.02^*^	0.000001	128.29^*^	0.000001	1.11	0.4062
	*Hcrtr2*	Hypocretin (orexin) receptor 2	0.45^*^	0.0018	0.66^*^	0.0034	1.44	0.0015	1.00	0.9990
	*Jun*	Jun oncogene	0.72	0.0010	0.60^*^	0.0105	0.94	0.1465	0.99	0.5810
	*Junb*	Jun B proto-oncogene	0.74	0.0191	0.36^*^	0.000031	1.45	0.0022	1.51^*^	0.0091
	*Myc*	Myelocytomatosis oncogene	0.59^*^	0.0005	0.35^*^	0.0003	1.75^*^	0.0288	1.83^*^	0.0015
	*Npffr1*	Neuropeptide FF receptor 1	0.21^*^	0.0031	0.16^*^	0.0027	3.09^*^	0.0004	1.66^*^	0.0200
	*Nsf*	N-ethylmaleimide-sensitive factor	0.61^*^	0.0013	0.53^*^	0.0003	1.30	0.0091	1.26	0.0378
	*Prlhr*	Prolactin releasing hormone receptor	0.38^*^	0.0233	0.13^*^	0.0083	2.51^*^	0.000022	1.04	0.4964
	*Ptgs2*	Prostaglandin-endoperoxide synthase 2	1.58^*^	0.0012	1.34	0.0097	0.93	0.0262	1.05	0.1204
The number of genes whose mRNA level changed			29		41		17		9	
			45				22			

*The mRNA levels of genes in the control group are taken as the baseline*.

* *and the bold values denote changes in the expression by 1.5 times or more, with a significance level p ≤ 0.05*.

For 23 genes whose expression changed 1 h after administration of either compound, a decrease in the mRNA level was observed. The effect was especially pronounced for three genes: *Gabre* (20 and 16.7 times for Selank and GABA, respectively), *Gabrq* (20 and 20 times for Selank and GABA, respectively), and *Hcrt* (25 and 50 times for Selank and GABA, respectively). An increase in mRNA level was noted only for two genes; the mRNA level of *Drd3* increased 3.4 and 2 times after Selank and GABA administration, respectively, and the mRNA level of *Gabrb3* increased 1.6 and 2.1 times after Selank and GABA administration, respectively.

Of the 16 genes whose expression changed only after GABA administration (1-h time point), 11 showed reduction in mRNA expression, in particular *Junb* and *Gabrd* (2.8 and 2.7 times, respectively). For the five genes *Birc5, Gabra1, Gabrb1, Gabrg3*, and *Glul*, the increase in mRNA level was no greater than two times compared with that of the control group. The genes whose expression changed only after Selank administration (1-h time point) were characterized by an increase in mRNA level, although not more than 2 times; the mRNA levels of *Drd2* and *Ptgs2* increased 1.6 times, and those of *Drd1a* and *Slc6a13* increased by 2 times.

A different pattern was observed 3 h after Selank or GABA administration. Expression changed in only 22 of the 77 genes selected for the analysis. The mRNA levels of 13 genes (*Abat, Adcy7, Ccnd1, Drd5, Gabre, Gabrq, HcRt, Htr3a, Prlhr, Slc32a1, Slc6a1, Slc6a11*, and *Slc8a3*) changed only after Selank administration. The mRNA levels of five genes (*Cx3cl1, Drd1a, Drd3, Gabra6*, and *Junb*) changed only after GABA administration. The mRNA levels of four genes (*Adora2a, Htr1b, Myc*, and *Npffr1*) changed after administration of either compound (Table 1). Level of mRNA increased for all genes except for *Drd3*, whose mRNA level decreased 1.8 times after GABA administration.

Similar to the pattern seen 1 h after Selank administration, at 3 h after administration of this compound, the most pronounced changes in gene expression were observed for *Hcrt, Gabre*, and *Gabrq*. At 3 h after Selank administration, the mRNA levels of these genes increased 128.3, 16.1, and 13.3 times, respectively. The mRNA level increased significantly 3 h after GABA administration for only one gene, *Gabra6*, whose mRNA level increased 7.6 times.

**Figure 1:**
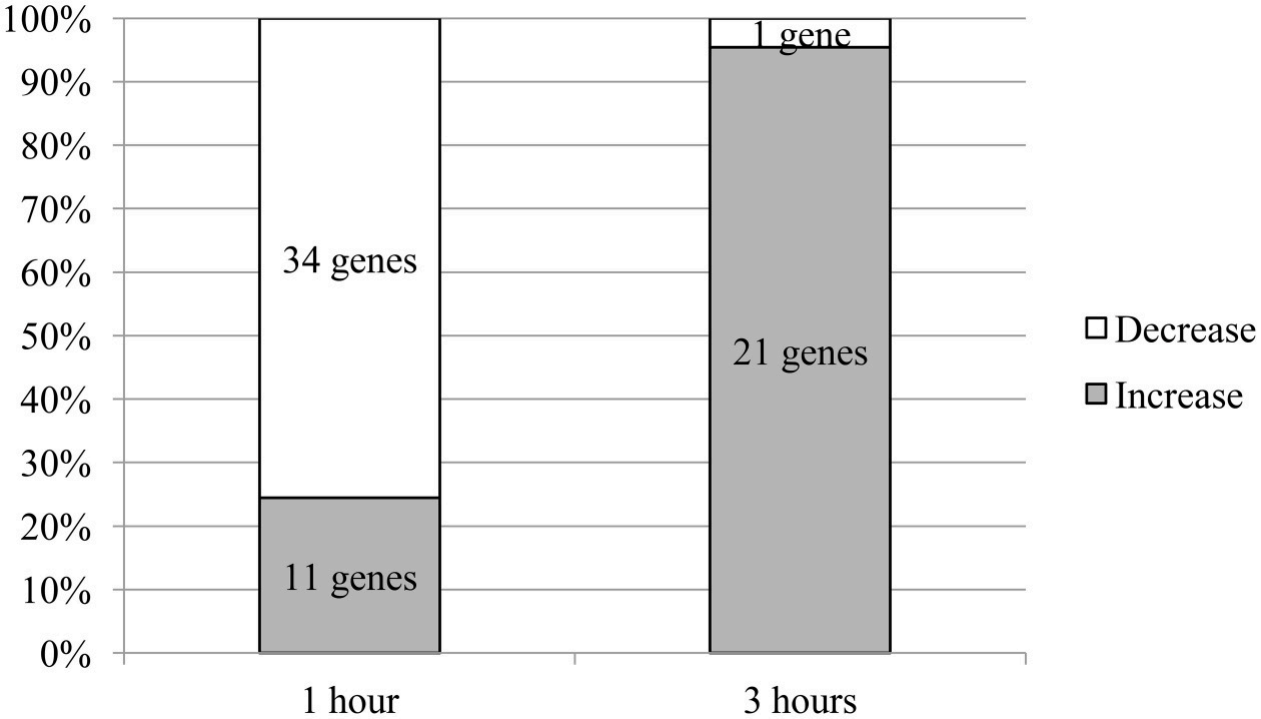
**The ratio of the number of genes with altered mRNA levels**. The proportions shown were normalised to the total number of genes with changed mRNA expression.

The aggregate analysis of all statistically significant data showed that the total number of genes whose mRNA level changed 1 h after administration of the test compounds (45 genes) was more than twice that of the genes whose expression changed after 3 h (22 genes). Most of the genes (76%) showed a decrease in mRNA level 1 h after administration of the test compounds. By contrast, the mRNA level increased in nearly almost all genes (95%) whose expression changed 3 h after administration of the test compounds (Figure 1).

Although 1 h after GABA administration, mRNA level changed in a substantial number of genes (53%, 41 of 77 genes), 3 h after GABA administration, the number of genes whose expression changed was reduced to only 9 (12%). In contrast to GABA, Selank affected the expression of fewer genes 1 h after the administration (38%, 29 of 77 genes), but the reduction in the number of genes whose expression changed 3 h after Selank administration was smaller (22%, 17 of 77 genes).

**Figure 2:**
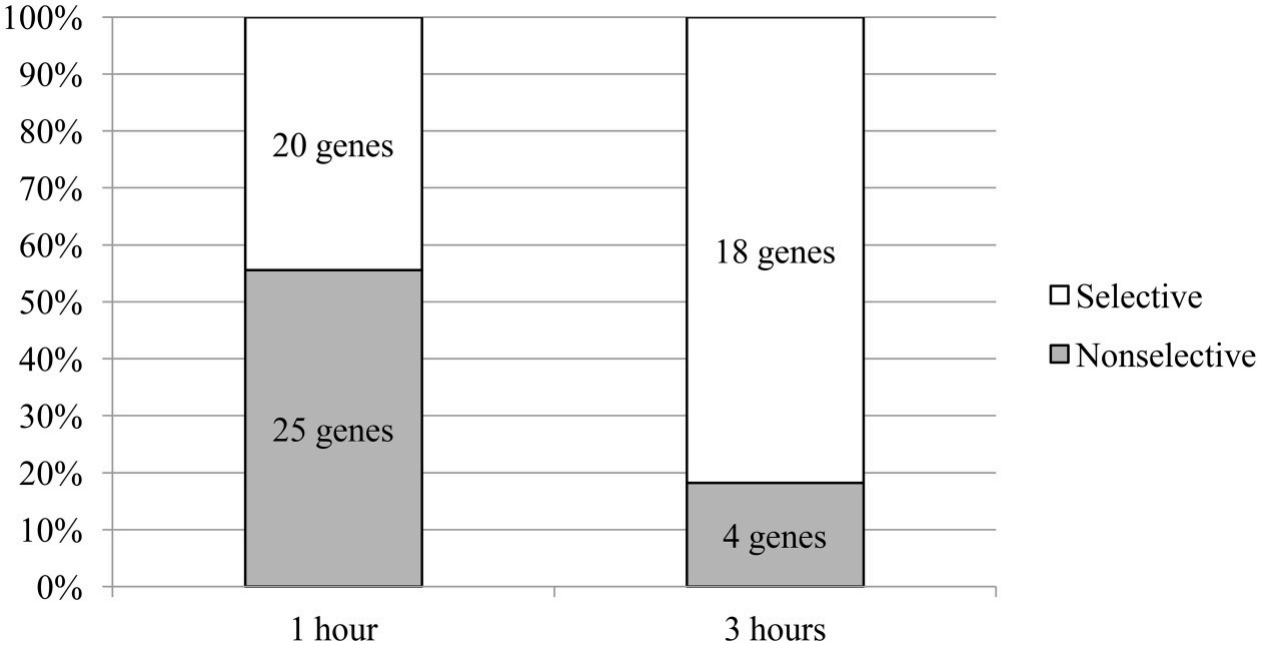
**The ratio of the number of genes that change levels of mRNA in the selective and nonselective action of the compounds**. The proportions shown are relative to the total number of genes that change the level of mRNA in the appropriate time period.

Another interesting feature was revealed: at the 1-h time point; 56% of the genes exhibited changed expression regardless of the test compound, whereas the change in expression was selective for one compound for 44% of the genes. A different pattern was seen at the 3 h-time point; only 18% of the genes exhibited changed expression regardless of the test compound, whereas the change in expression of 82% of the genes was selective for one compound (Figure 2).

Correlation analysis was performed for those genes whose mRNA level changed significantly after administration of the test compounds. A positive correlation was observed between the changes in gene expression within 1 h after administration of Selank or GABA (*r* = 0.86; *p* ≤ 0.05). By contrast, a negative correlation was observed between the changes in gene expression at the 3-h time-point after the administration of Selank or GABA (*r* = −0.39; *p* ≤ 0.05).

## Discussion

Numerous clinical studies have shown that Selank has strong antianxiety and neuroprotective effects in the treatment of generalized anxiety disorders. The clinical effects of Selank are similar to those of the classical antianxiety medications such as benzodiazepines, which are allosteric modulators of GABA_A_ receptors and increase the inhibitory action of GABA (Seredenin et al., 1990, 1998). This suggests that the molecular mechanism of action of Selank arises from its ability to affect GABA receptors.

To test this hypothesis, we assessed the changes in the mRNA levels of 84 genes encoding proteins related to the functioning of the GABAergic system, as well as those of proteins involved in neurotransmission in the frontal cortex of rats 1 and 3 h after the administration of Selank or GABA.

Our results suggest that Selank is able to directly influence the expression of genes involved in neurotransmission in nerve cells, and similar changes in the expression of these genes are also observed when GABA is administered. This is supported by the strong positive correlation between the changes in the expression of 45 genes 1 h after the Selank or GABA administration. However, the match between the expression profiles of these genes is not perfect. Thus, in addition to the 25 genes affected by either of the test compounds, GABA but not Selank affected the mRNA level of another 16 genes 1 h after administration. This difference in the number of genes whose expression changed suggests that Selank acts not directly through the center of the specific binding of GABA, but rather allosterically by altering the affinity of the GABA receptor for GABA. Previously, it was shown that Selank is able to affect the specific binding of GABA to its own receptors, which may be caused by modulating properties of regulatory peptide, which apparently change the affinity of endogenous ligands under the influence of Selank on the receptor (V'Yunova et al., 2014). We can assume that the observed similarity of expression profiles of our study genes after administration of Selank and GABA partly confirms the hypothesis about the possible effect of the peptide through the regulation of the activity of GABA_A_ receptors.

We note that, compared with the 1-h time point, there was a sharp decrease at 3 h in the number of genes whose expression changed after exposure to GABA. The large number of genes whose mRNA level changed 1 h after GABA administration suggests that rapid effects were caused by binding of GABA to GABA_A_ receptors. This binding leads to the opening of ion channels in the nerve cell membrane of and the subsequent entry of chloride ions through the channels (MacDonald and Olsen, 1994). The absence of significant changes 3 h after GABA administration may be associated with a gradual decrease in the activity of the major elements of the GABAergic system. In contrast to GABA, a significant reduction in the number of genes whose expression changed 3 h after Selank administration was not observed. This suggests that Selank activates alternative processes, which cause delayed changes in the expression of certain genes that do not relate directly to the operation of the GABAergic system but at the same time contribute to the activation of certain genes involved in its operation.

Interestingly, the mRNA levels of four genes (*Drd1a, Drd2, Ptgs2*, and *Slc6a13*) altered only under the influence of Selank at the 1-h time point. Intriguing results were obtained in relation to *Slc6a13*, which encodes the low-affinity transporter of the GABA GAT-2. GAT-2 plays a key role in peripheral mechanisms involved in the work of GABAergic system and is also responsible for the redistribution and metabolism of drugs, which can affect the operation of the GABAergic system (Schlessinger et al., 2012). Thus, activation of the carrier only after exposure to Selank suggests the presence of an alternative path of action of the peptide on the distribution of peripheral GABA. *Drd2* and *Drd1a* encode dopamine receptors, which are associated with G-protein and are involved in the regulation of adenylate cyclase activity, thereby mediating intracellular signal transduction (Beaulieu and Gainetdinov, 2011). The activation of *Drd5* expression at the 3-h time point was observed only for Selank. *Drd5* encodes the dopamine receptor, which plays a key role in the formation of memory and learning processes by ensuring long-term potentiation (Beaulieu and Gainetdinov, 2011). Activation of this gene by Selank at early and later times suggests an ability of the peptide to influence processes involved in synaptic plasticity and thereby render nootropic action. It has been shown previously that Selank modulates dopamine and serotonin receptors, which play a role in the stimulation of mental activity and in the pathogenesis of anxiety (Meshavkin et al., 2006).

Of particular interest is the significant change in the mRNA level of *Hcrt* 3 h after Selank administration. This gene encodes a precursor of orexins and is involved in the regulation of the balance between sleep and wakefulness (Ohno and Sakurai, 2008). Kolomin et al. have shown that the mRNA level of the *Hcrt* increases after a single administration of Selank (Kolomin et al., 2013). The presence of this significant effect of Selank suggests that the peptide has an active effect on the balance between sleep and wakefulness, and that the change in the expression of *Hcrt* may be in the foundation of the normalizing effect Selank has on the balance of sleep patterns in patients with general anxiety disorders. The observed changes may also explain the lack of hypnosedative action of Selank, which is common for classical benzodiazepines, because of the shift in the balance toward wakefulness.

Thus, our obtained data indicate that, like other peptide drugs, Selank is characterized by its complex effects on nerve cells. One of its possible molecular mechanisms is associated with allosteric modulation of the operation of the GABAergic system. At the same time, Selank may act through other systems such as the dopamine and serotonergic systems. Further research is needed to identify all aspects of the mechanisms of action of Selank and the effects of this peptide on various neurotransmitter systems in different models.

## Author contributions

AV, TK, LA performed the experimental work. AV, MS, and PS undertook all statistical analyses and helped with their interpretation. PS, MS designed the study. AV, MS wrote the first draft of the manuscript. MS and PS contributed to the final writing of the manuscript. SL, NM was involved in revising the manuscript critically for important intellectual content. All authors contributed to and have approved the final manuscript.

### Conflict of interest statement

The authors declare that the research was conducted in the absence of any commercial or financial relationships that could be construed as a potential conflict of interest.
